# The Role of Socioeconomic Status in Adherence to the Mediterranean Diet and Body Mass Index Change: A Follow-Up Study in the General Population of Southern Croatia

**DOI:** 10.3390/nu13113802

**Published:** 2021-10-26

**Authors:** Ajka Pribisalić, Romana Popović, Fiorella Pia Salvatore, Maja Vatavuk, Marija Mašanović, Caroline Hayward, Ozren Polašek, Ivana Kolčić

**Affiliations:** 1 Department of Public Health, University of Split School of Medicine, Šoltanska 2, 21000 Split, Croatia; ajka.pribisalic@mefst.hr (A.P.); opolasek@mefst.hr (O.P.); 2 NUTRITIUS—Nutrition Counseling, Primorska 30, 20000 Dubrovnik, Croatia; nutritius.romana@gmail.com; 3 Department of Economics, University of Foggia, 71121 Foggia, Italy; fiorellapia.salvatore@unifg.it; 4 Department of Cardiac Surgery, University Hospital of Split, 21000 Split, Croatia; majavatavuk1@gmail.com; 5 Department for Social Medicine, Division for Health Promotion, Public Health Institute of Dubrovnik Neretva County, Dr. A. Šercera 4a, pp 58, 20001 Dubrovnik, Croatia; marija.masanovic@zzjzdnz.hr; 6 MRC Human Genetics Unit, Institute of Genetics and Molecular Medicine, University of Edinburgh, Edinburgh EH8 9YL, UK; caroline.hayward@ed.ac.uk; 7 Algebra LAB, Algebra University College, Ilica 242, 10000 Zagreb, Croatia

**Keywords:** Mediterranean diet, adherence, BMI, socioeconomic status

## Abstract

The Mediterranean diet (MD) is one of the most healthful dietary patterns, beneficial for humans and the environment. However, the MD has recently exhibited a declining trend, especially in younger and less affluent people. This study investigated the association between socioeconomic indicators and adherence to the MD in 4671 adult subjects from Dalmatia, Croatia (age range 18–98 years; 61.9% were women). Additionally, in the follow-up we examined the change in adherence to the MD and in BMI (subsample, N = 1342; 62.5% were women; mean follow-up time of 5.8 years). The adherence to the MD was based on the Mediterranean Diet Serving Score (range 0–24 points, cut-off value ≥ 14 points), with a prevalence in the overall sample of 28.5%. Higher odds of adherence to the MD were recorded in women, older subjects, and those with higher level of objective material status, while it was less likely in the period after economic crisis of 2007–2008. Additionally, we detected no change in adherence to the MD in the follow-up subsample (−8.5%, *p* = 0.056), but there was an increase in BMI (+6.5%, *p* < 0.001). We recorded an increase in adherence for nuts (+127.5%), sweets (+112.6%), red meat (+56.4%), and wine (+50.0%), unlike the reduction in adherence for vegetables (−35.1%), fish (−23.4%), white meat (−11.6%), cereals (−10.9%), and dairy products (−9.6%). Similar results were obtained across all quartiles of objective material status. Over time, the absolute change in the MD score was positively associated with female gender, age, higher education, and moderate physical activity, but it was negatively associated with adherence to the MD at baseline. BMI change was positively associated with female gender, and negatively with initial BMI, initial adherence to the MD, and MD change. Our findings point towards a less than ideal adherence to the MD in the general population of southern Croatia, and identify important characteristics associated with adherence change over time, informing necessary interventions aimed at increasing MD uptake.

## 1. Introduction

Unhealthy lifestyle and unhealthy diet in particular are among the foremost public health challenges, with as many as 11 million deaths globally being attributable to suboptimal diet in 2017 [1]. The leading global dietary risk factors for death and disability were high sodium intake, low intake of whole grains, and low intake of fruits [1]. These are highly preventable risk factors that could be addressed by adopting scientifically proven healthy diets at the population level.

One model of healthy eating that is particularly well described in the literature is the Mediterranean diet (MD), which is especially healthful compared to a more westernized dietary pattern [2]. MD is characterized by a high intake of plant-based foods, such as daily intake of vegetables, fruit, whole grains, olive oil, nuts and seeds, and weekly intake of dairy, fish and legumes, alongside frugal use of meat, eggs and sweets [3]. There is a large body of evidence showing that adherence to the MD can preserve human health, with the extra bonus of ensuring environmental sustainability [4,5]. The health benefits of MD are numerous [6,7]. The most important positive effects include reduced all-cause mortality [6,8,9], primary prevention of cardiovascular disease [10], lower cancer incidence and mortality [6,11], reduced risk for development of type II diabetes [12,13,14], obesity and metabolic syndrome [13]. Benefits of MD also include safeguarding of mental health, such as better cognitive performance with higher adherence to the MD [15], reduced risk of depression and cognitive impairment [16], lesser mental distress [17], and overall better health-related quality of life [18,19]. Moreover, MD was even shown to be an efficient treatment strategy for major depressive episodes [20].

Regardless of these and other health benefits of MD and other traditional diets, global nutrition transition caused by modernization and increased incomes has resulted in deviation from traditional plant-based diets towards higher intake of animal-source food, added sugar and vegetable oils [21]. An analysis of the supply of the most important food components of the traditional MD in several Mediterranean countries has revealed that these countries have experienced a process of Westernization during the period from 1961 to 2001, which was especially pronounced in the European countries of the Mediterranean basin [22].

Major constitutional components of the MD, such as fruit, vegetables, olive oil and fish are still present within the dietary pattern, but the discrepancies between Mediterranean countries and regions have started to emerge more consistently [23]. For example, MD decline was observed in Malta, unlike Sardinia, which was accredited to “modernity and improved living conditions, enhanced commercial availability and increased diversity of food preparation” [24]. However, an overall declining trend in adherence to the MD has been previously demonstrated in many Mediterranean countries [25,26,27], especially in younger generations [28,29,30,31,32,33]. On the other hand, some countries have experienced an increase in adherence to the MD among adolescents, such as Israel, where increased consumption of fruits, vegetables, cereals, dairy products, and decreased negative eating behaviors were recorded in 2016 compared to 2003 [34].

Besides the greater convenience of a diet relying on processed foods and ready-to-eat fast food, saving time and effort, these foods are also readily available in our modern urbanized environments. They are appetizing and tasty, and they may be cheaper than whole foods. Indeed, the question of a monetary cost behind the Mediterranean dietary pattern has been previously investigated. Some of these previous studies have shown that greater adherence to the MD was associated with a higher dietary cost [35,36,37], especially if it is compared to a Western dietary pattern [38]. Therefore, it is not surprising to consistently find that the lowest-income households had the lowest adherence to the MD and the highest obesity prevalence [39]. However, it was shown that a higher educational status could exhibit a mitigating effect on poorer diet in lower income countries [40]. These findings demonstrate a complex interplay between different socio-economic determinants and dietary habits.

Furthermore, since we can define socio-economic status (SES) by using several characteristics, it may be challenging to disentangle the main SES contributor to various health outcomes. SES characteristics include objective indicators, such as attained level of education, profession, employment/unemployment status, income, and the subjective perception of one’s wealth compared to other people within the same community. Despite this complexity, the impact of SES on dietary pattern is undeniably important. This effect was summarized nicely in a recent paper stating that people “who are better off consume healthier diets than those less well-to-do” [41]. Unfortunately, a clear link between low SES, poor health and obesity was also recognized [41], making it a double priority in terms of the need for effective public health interventions and more broader political, economic and societal interventions against inequalities. In this context, the MD and the overall Mediterranean lifestyle could lend itself “as the most appropriate regime for disease prevention, a sort of complete lifestyle plan for the pursuit of healthcare sustainability” [41]. Indeed, it was consistently shown that people more adherent to the MD had more favorable anthropometric indicators. For example, a large cohort study with a mean of 12 years of follow-up showed that people with high adherence to the MD had a lower risk of becoming overweight/obese, experienced lesser 5-year change in waist circumference, and had lower 5-year weight change in the case of normal weight at baseline [42]. Additionally, MD was found to be more effective in long-term weight loss (over two years of follow-up) in patients with metabolic syndrome than a prudent control diet [43]. It was also found that in older Mediterranean individuals with excess weight, those subjects who desired higher weight loss actually had lower adherence to the MD and higher prevalence of obesity [44]. Hence, MD could serve as a good model for both keeping weight stable across life, and for sustainable weight loss [45].

There is a paucity of studies investigating the trend in adherence to the MD in Croatia. In general, based on geographical location and cultural heritage, the population of the Adriatic region of Croatia is adherent to the MD and the Mediterranean lifestyle [46]. Additionally, Croatia was one of the countries that supported in the inclusion of MD on the UNESCO’s Representative List of the Intangible Cultural Heritage of Humanity [47]. However, the role of different socio-economic characteristics in the MD pattern and BMI change in Croatia has been only marginally investigated. It was previously shown that a lower education level was associated with lower adherence to the MD in the population of southern Croatia, while the overall prevalence of adherence to the MD was also rather low [31]. On the other hand, Croatia is heavily encumbered with non-communicable diseases [48], and ranks high among the leading countries in Europe regarding the prevalence of overweight and obesity, with 58% of the adult population being affected [49]. This undesirable trend is present even in young children, with as many as 35.9% of 7–9 year-olds being overweight or obese [50]. Therefore, our aim was to estimate the temporal trend in adherence to the MD and the contribution of several socio-economic factors in the changing pattern of the MD and BMI in a follow-up study including a large sample from Dalmatia, Croatia.

## 2. Materials and Methods

### 2.1. Study Participants

This study included 4988 subjects, between 18 and 98 years old, from several settlements in Dalmatia, Croatia, upon their initial enrolment within the “10,001 Dalmatians” study [51], while the follow-up data were available for 1342 subjects. The main objective of the “10,001 Dalmatians” study was to explore genetic and environmental risk factors by creating a biobank in the isolated populations of the Adriatic islands.

Chronologically, the initial field study was performed during 2003 and 2004 on the Island of Vis (N = 1029). An additional 969 subjects were enrolled from the Island of Korčula in 2007 (the Town of Korčula and surrounding settlements), followed by 1012 subjects from the City of Split in 2008–2009. Finally, 857 subjects were included in 2013 from the villages of Smokvica and Čara, situated in the central part of the Island of Korčula, and 1121 subjects were included during 2014–2015 from the towns of Blato and Vela Luka on the western part of the Island of Korčula.

The initial population-based convenient sampling approach employed personal invitations by general practitioners, postal invitations, local media and support from other local stakeholders, namely local governments and priests. Only subjects older than 18 were eligible to participate in the study, without any other restrictions or exclusion criteria. After being formally informed of the study objectives, subjects signed the informed consent before the enrolment.

The field-based follow-up data collection was performed in 2011 for the subjects from the Island of Vis (N = 482, response rate 46.8%, mean follow-up of 7.5 years). In 2013 we collected follow-up data for the subjects from the Town of Korčula who were initially included in 2007 (N = 366; 37.8%; mean follow-up of 5.3 years), and in 2012–2013 for the subjects from the City of Split (N = 494; 48.8%, mean follow-up of 4.4 years). The main reason for the different follow-up times between study sites is the use of an open cohort sampling approach; this inevitably led to a different amount of time that each participant could be followed for. Subjects from Smokvica, Čara, Blato and Vela Luka (N = 1978) were not included in the follow-up due to their initial inclusion in 2013–2015, after which no additional data collections were done within the “10,001 Dalmatians” study.

The study was approved by the Ethical Committee of the University of Split School of Medicine.

### 2.2. Data Collection and Measurements

Trained nurses and medical doctors performed anthropometric measurements and collected clinically relevant information using the standard operating procedures at the newly established study site in each location. Individual medical histories were taken, together with an extensive self-administered questionnaire (including demographic characteristics, detailed socioeconomic status, dietary habits, smoking habits, alcohol consumption, and physical activity). Elderly people and those with any disabilities were offered assistance during surveying by a team of nine trained surveyors.

Medical records or subjects’ responses were used to extract relevant medical history information, including previous diagnoses and the usage of medications for hypertension, diabetes, coronary heart disease (CHD), cerebrovascular insult (CVI), cancer, bipolar disorder, hyperlipidemia and gout.

### 2.3. Socioeconomic Status

Socioeconomic status was assessed during the initial data collection using three determinants: education, subjective material status, and objective material status. Education was categorized into three groups in order to correspond to the Croatian educational system [52]. The three groups were constructed according to the number of completed years of schooling, which corresponded to primary education (≤8 years of schooling), secondary (high school level with 9–12 years of schooling), and higher education (≥13 years). Only 17 subjects reported being students during the initial data collection, and they were automatically included in the higher education group of education.

Subjective material status was assessed based on the participant’s perception of her/his material status in comparison to other people in their community. Possible responses on this question were ‘much worse than the average’, ‘somewhat worse than the average’, ‘the same as others’, ‘better than the average’, ‘much better than the average’. These responses were grouped into three categories for easier interpretation: worse than average (responses ‘much worse than average’ and ‘somewhat worse than average’), average (‘the same as others’), and better than average (including answers ‘better than average’ and ‘much better than average’).

Assessment of objective material status was obtained based on the possession of 16 material items or goods, including heating system, wooden floors, video/DVD recorder, telephone, computer, two TVs, freezer, dishwasher, water supply system, flushing toilet, bathroom, library with more than 100 books, paintings or other art, a car, vacation house or second apartment, and boat, as in our previous study [53]. The sum of those items in the subject’s possession indicated the wealth of the subject. Based on the distribution of these wealth scores, quartiles of objective material status were formed: the first quartile with values ≤ 8, second quartile with 9–10, third quartile with 11–12, and fourth quartile with values 13–16, as in our previous study [52].

Formal income was not taken into account due to the long period of observation included in this study (from 2003–2015), during which many economic and social changes happened in Croatia, including the financial crisis of 2007–2008. In order to take this into account in our analysis, we have introduced the variable for the recession period (before/after), denoting it as having started in our target population after 2008 (and including subsequent years).

### 2.4. Mediterranean Diet Assessment

Assessment of the Mediterranean dietary pattern was based on the food frequency questionnaire (FFQ), which was adjusted for application in the population of Dalmatia. There were 55 questions on commonly consumed foods, with 6 possible responses (every day, 2–3 times a week, once a week, once a month, rarely, and never), investigating the frequency of consumption of olive oil and other fats, milk and dairy products, vegetables, fruits, nuts, legumes, various meats, fish and sea foods, eggs, sweets, potatoes, rice, pasta, and bread [31,52]. Mediterranean diet adherence was assessed using the Mediterranean Diet Serving Score (MDSS), which incorporates 14 typical food groups representing the modern MD pyramid: fruit, vegetables, cereals, potatoes, olive oil, nuts, dairy products, legumes, eggs, fish, white meat, red meat, sweets, and fermented beverages—namely wine [54]. MDSS and adherence to the MD were calculated as described previously [31,52], and subjects were classified as adherent to the MD in case they had reached ≥14 points (the range was 0–24 points, with no negative points). MDSS requires a daily intake of vegetables, fruit, olive oil, and cereals (intake of each group is awarded with three points for two or more servings a day). Daily intake is encouraged for nuts and dairy products (each group is awarded with two points for one or more servings a day), and for wine (one or two glasses per day, awarded with one point) [54]. The remaining food groups are awarded with one point. Namely, red meat and sweets should be among the less frequently eaten foods (two or less servings per week), while potatoes, legumes, eggs, fish, and white meat should be consumed weekly. This questionnaire was also validated for use in the Croatian population in the short form [55].

We have excluded 317 subjects from the analysis due to missing values in the FFQ and the inability to calculate the MDSS at baseline.

### 2.5. Lifestyle Characteristics 

Besides diet and socioeconomic factors, we assessed other lifestyle indicators, such as smoking and physical activity. According to smoking status, we divided subjects into current smokers, ex-smokers (those who reported they ceased smoking more than a year ago), and those who had never smoked. Assessment of physical activity included activity during both the working part of the day and the leisure part of the day. Those subjects who reported hard intensity labour or other high-intensity activity during either part of the day were considered as intensively physically active. Subjects who reported moderate intensity of physical activity in either part of the day were considered moderately active, while all others reporting either sitting or light physical activity in both parts of the day were considered as having light physical activity.

Additionally, body mass index (BMI) was calculated using measured height and weight. BMI was divided into three categories, representing subjects with normal body weight (from 18.5 to 24.9 kg/m^2^), overweight (25.0 to 29.9 kg/m^2^), and obese subjects (≥30.00 kg/m^2^).

### 2.6. Statistical Analysis

All categorical variables were described using absolute numbers and percentages. All numerical variables were described using median and interquartile range (IQR), due to non-normal distribution, which was tested using the Kolmogorov–Smirnov test. The χ^2^ test was used to examine the differences between groups for categorical variables and Kruskal–Wallis for numerical variables. We additionally investigated the differences between included subgroups; Mann–Whitney U test was used for pairwise comparison of numerical variables and χ^2^ for categorical variables.

Univariate and multivariate logistic regression analysis (enter method) were used to assess the association between three SES characteristics (education level, subjective material status, objective material status) and overall adherence to the MD (MDSS ≥ 14 points) at baseline. Additionally, multivariate logistic regression analysis was used for assessing predictors for adherence for each of the 14 MD food groups within the MDSS scoring system. All multivariate models included age, sex, place of residence, number of chronic diseases diagnosed previously, smoking, physical activity, and BMI as confounding factors. There were only 53 subjects in the baseline sample with BMI less than 18.5 kg/m^2^, and we have excluded them from the regression analysis due to the small sample size of the group. Additionally, in order to control for the potential confounding effects of the recession of 2007–2008, we included a variable denoting the time period of data collection as being either before or after the recession period in all of the regression models. All of the included covariates were entered as categorical variables to enable easier interpretation of the results. Odds ratios (OR) and 95% confidence intervals (CI) were provided for both univariate and multivariate logistic regression models. Correlations between the three variables describing socioeconomic status were tested using the Spearman rank test, before using them together in logistic regression models; none of the Spearman’s rho values were higher than 0.401.

Linear regression models were used to assess the association between absolute change in MDSS and BMI across the follow-up period with different subjects’ characteristics. The main predictor variables were again the three SES characteristics (education level, subjective material status, and objective material status), and the models also included important confounding variables: age, follow-up time, sex, place of residence, number of chronic diseases diagnosed previously, smoking, physical activity, BMI at baseline, and MDSS at baseline. Additionally, the model with BMI change during the follow-up as an outcome variable also included the MDSS absolute change during the follow-up as a covariate.

The change in the prevalence of the adherence to the MD and each of the MDSS food groups between baseline (*t*_0_) and the follow-up time period (*t*_1_) was assessed by calculating the percent change, using the following formula:

(1) $$MD\;adherence\;{\left(\%\right)}_{change}=\frac{\mathrm{MD}\;\mathrm{adherence}{\left(\%\right)}_{t_{1}}-\mathrm{MD}\;\mathrm{adherence}{\left(\%\right)}_{t_{0}}}{\mathrm{MD}\;\mathrm{adherence}{\left(\%\right)}_{t_{0}}}*100,$$

Additionally, the absolute change in MDSS score and BMI between baseline (*t*_0_) and the follow-up time period (*t*_1_) was calculated using the following formulas:

(2) $$MDSS_{change}={\;\mathrm{MDSS}}_{t_{1}}-{\;\mathrm{MDSS}}_{t_{0}}$$

(3) $$BMI_{change}={\;\mathrm{BMI}}_{t_{1}}-{\;\mathrm{BMI}}_{t_{0}}$$

The Wilcoxon signed-rank test and McNemar test were used to compare the differences between paired data for repeated measurements (baseline vs. follow-up).

The significance level was set at *p* < 0.05. All statistical analyses were carried out using IBM SPSS Statistics v21 (IBM, Armonk, NY, USA).

## 3. Results

The analysis included 4671 subjects in total (Table 1). Subjects from the Island of Vis were on average older, less educated, and had the highest average BMI (median of 27.08; IQR 6.05). The median MD adherence score (MDSS) was the lowest in subjects from the Island of Korcula (11 out of 24 points; IQR 6), and it was slightly higher in both subjects from the City of Split and the Island of Vis (median 12; IQR 5). Significant differences in median MDSS score were also recorded between settlements and according to age groups (Table 1). A wide range of adherence to MDSS components was present, ranging from as low as 2.7% for nuts in subjects from Vis, and up to 97.4% adherence for cereals in the same group (Table 1).

Less than half of all of the subjects were compliant with the daily requirement for vegetable intake (lowest on Korčula; 37.0%), while it was a little better for intake of fruit (lowest on Korčula; 52.8%), and olive oil (lowest on Vis; 57.9%). Only 22.3% of subjects from the Island of Korčula, 25.3% from the Island of Vis and 26.8% from the City of Split adhered to the daily dairy products consumption requirement, which was similar for wine (17.3–20.2%). Consistently, the best adherence was recorded for cereals, and the lowest for nuts (Table 1). A total of 1332 subjects (28.5%) were considered as being adherent to the MD pattern in the overall sample. The lowest prevalence was recorded for subjects from the Island of Korčula (26.8%), followed by those from the City of Split (30.4%), and the Island of Vis (31.1%). There was a significant difference in the prevalence of adherence to the MD according to age groups and place of residence, and a significant result was obtained for the comparison between subjects from Vis and Korčula (*p* = 0.026; Table 1).

Logistic regression analysis revealed several characteristics that were strongly associated with adherence to the MD throughout the entire sample (Table 2). Women presented higher odds of adherence compared to men (OR = 1.85, 95% CI 1.58–2.17, *p* < 0.001), while the oldest age group had 3.81-fold higher odds of adherence compared to the youngest subjects (95% CI 2.83–5.12, *p* < 0.001; Table 2). In the fully adjusted model, subjects from the Island of Korčula presented with higher odds of adherence compared to the subjects from the City of Split (OR = 1.63, 95% CI 1.31–2.102, *p* < 0.001). Education level and subjective material status were not associated with adherence to the MD in the adjusted model, unlike objective material status. The wealthiest subjects according to the objective material status (those in the fourth quartile of distribution) were almost twice as likely to be adherent to the MD, compared to subjects in the lowest quartile (OR = 1.93, 95% CI 1.53–2.43, *p* < 0.001). Subjects in the second and third quartile of objective material status also had greater odds of being adherent to the MD (Table 2).

Subjects who never smoked and ex-smokers presented with higher odds of adherence to the MD, compared to current smokers (OR = 1.36, 95% CI 1.13–1.63, *p* = 0.001; OR = 1.40, 95% CI 1.14–1.71, *p* = 0.001, respectively). Subjects with higher levels of physical activity were also more likely to be adherent to the MD (Table 2). BMI and diagnosis of chronic diseases were not associated with adherence to the MD. The study period was statistically significantly associated with adherence to the MD, in a way that MD adherence was less likely in the period after the recession (OR = 0.31, 95% CI 0.25–0.38, *p* < 0.001; Table 2).

The fully adjusted regression model yielded a good data fit (Hosmer and Lemeshow *p* = 0.304; Nagelkerke R2 = 0.100).

Determinants of adherence to MD food components are shown in Supplemental Table S1. Women were more likely to be adherent to the recommended intake of fruit, vegetables, olive oil, nuts, diary, and red meat, but they were less likely to be adherent to the eggs and wine intake MD recommendations compared to men (women most commonly abstained from alcohol intake). Older subjects had higher odds for meeting the recommendations for fruit, vegetables, cereals, olive oil, nuts, fish, red meat, sweets, and wine intake, but lower odds for potatoes and eggs adherence compared to the youngest group of subjects. The highest level of education was associated with lesser adherence to the MD guidelines for intake of cereals, olive oil, legumes, fish, and white meat, in contrast to a higher adherence to appropriate intake of dairy products, potatoes and red meat compared to subjects with the lowest level of education (Supplemental Table S1). Subjective material status was less associated with MD food components intake, unlike the objective material status. Compared to subjects in the lowest quartile of objective material status, subjects belonging to higher quartiles presented with an increasing trend of compliance with fruit, vegetables, olive oil, and fish intake recommendations, but also with a decreasing compliance for the intake of red meat and sweets (Supplemental Table S1).

Obese subjects (BMI 30 ≥ kg/m^2^) were 34% more likely to adhere to recommendations for sweets, but also 30% less likely to adhere to recommendations for cereals intake, and 42% less adherent for nuts.

The study period after the recession was associated with 68% decreased odds for adherence to vegetables intake recommendations, 55% decreased odds for cereals adherence, 50% for fruit, 49% for fish, 47% for legumes, 36% for dairy products, 31% for potatoes, and 29% decreased odds for adherence to olive oil intake. On the other hand, we recorded 39% increased odds for adherence to red meat and 23% increased adherence to sweets intake recommendation after recession (Supplemental Table S1).

**Figure 1:**
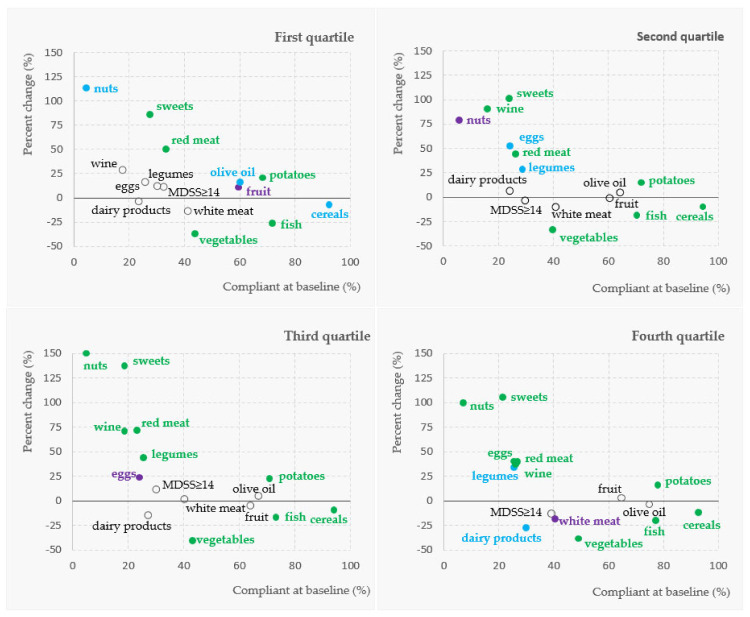
Change in adherence to the MD food components and the overall MD (MDSS ≥ 14 points), expressed as a percentage change from baseline to the follow-up, according to the objective material status category. Significant results at the level of *p* < 0.05 are denoted with the full circle (green < 0.001, blue < 0.01, purple < 0.05, McNemar test).

In order to assess the change in Mediterranean diet compliance over time, 1342 subjects were included in the follow-up study. A breakdown by four quartiles of the objective material status demonstrated significant changes in adherence for several MD food groups across the follow-up period (Figure 1). A distinct pattern of change was recorded, with the most prominent and significant decrease in adherence to the recommended intake of vegetables, followed by a decrease in fish and cereals recommended intake across all quartiles of objective material status (Figure 1). On the other hand, a significant increase in adherence for nuts was reported across all quartiles of material status (corresponding to increased intake), followed by an increase in sweets, potatoes and red meat (decreased intake), wine, legumes, and eggs adherence (increased intake). The exception was adherence to wine, legumes, and eggs recommendations in subjects within the lowest quartile of the objective material status, where these results were not significant. Based on such diverse results in individual MDSS food groups, the overall change in adherence to the MD was insignificant in all of the quartiles of objective material status (Figure 1). A similar result was obtained in the total group of subjects included in the follow-up, with a borderline insignificant decrease in adherence to the MD (by 8.5%; from 36.6% of adherent subjects at study baseline, to 33.5% in the follow-up; *p* = 0.056; Table 3). Furthermore, the highest overall increase in adherence was recorded for nuts (127.5%), and sweets (112.6%), followed by red meat (56.4%), and wine (50.0%). On the other hand, the most significant decrease in adherence was recorded for vegetables (−35.1%), followed by fish (−23.4%), white meat (−11.6%), cereals (−10.9%), and dairy products (−9.6%). At the same time, the average BMI had increased from 25.76 kg/m^2^ at baseline of the study to 27.44 kg/m^2^ at the follow-up time period (*p* < 0.001).

Linear regression analysis revealed several variables that were significantly associated with the MDSS change during the follow-up period (Table 4). MDSS change was positively associated with female gender (β = 0.41; 95% CI 0.00–0.83; *p* = 0.049), age (β = 0.05; 95% CI 0.03–0.06); *p* < 0.001), highest level of education (β = 0.71; 95% CI 0.07–1.36; *p* = 0.031), and with moderate physical activity (β = 0.72; 95% CI 0.27–1.16; *p* = 0.002). MDSS at baseline displayed a negative association with the MDSS change (β = −0.64; 95% CI −0.70–−0.58; *p* < 0.001), while BMI at baseline, smoking, chronic diseases, place of residence, objective and subjective material status were not associated with the absolute change in the Mediterranean Diet Serving Score. The regression model yielded a good data fit (Durbin–Watson = 1.994; Adjusted R2 = 0.280).

BMI change during the follow-up period was significantly associated with female gender, place of residence, BMI at baseline, MDSS at baseline and MDSS absolute change (Table 4). Women experienced higher odds for BMI increase compared to men (β = 0.33; 95% CI 0.06–0.60; *p* = 0.016), the same as subjects from the Island of Vis and Korčula compared to subjects from the City of Split (β = 0.86; 95% CI 0.18–1.54; *p* = 0.013, and β = 3.68; 95% CI 3.15–4.21; *p* < 0.001, respectively). BMI at baseline, MDSS at baseline, and MDSS change during the follow-up were all significantly negatively associated with the BMI change (β = −0.11; 95% CI −0.14–−0.07; *p* < 0.001, β = −0.07; 95% CI −0.12–−0.03; *p* = 0.001, β = −0.04; 95% CI −0.07–0.00; *p* = 0.041, respectively), while none of the socio-economic characteristics were associated with absolute BMI change. The regression model yielded good data fit (Durbin–Watson = 1.972; Adjusted R2 = 0.354).

## 4. Discussion

Our results demonstrated a rather low prevalence of adherence to the MD over the entire sample (28.5%), especially among younger individuals (14.0%). Subjects included in the follow-up had a higher adherence to the MD at baseline (36.6%), with a borderline insignificant decline at the end of the follow-up period (33.5%). On the other hand, BMI had increased on average by 6.5% in subjects available for follow-up.

Our result for MD prevalence was within the expected range, compared to the results from other Mediterranean countries and from Croatia. For example, findings from the literature vary anywhere between 14% of adherent people in Northern Italy [56] to 45% in Balearic Islands [57]. Our current study identified a slightly higher prevalence of adherence to the MD compared to our previous results, when we identified 23% of subjects as adherent to the MD [31]. This difference is due to a smaller sample and different period included in the previous study [31].

Unfortunately, many Mediterranean societies are moving away from their traditional dietary pattern, while some countries in Northern Europe and around the world are adopting a Mediterranean-like dietary pattern [25]. For example, previous studies have indicated a persistent moderate-to-weak adherence to the MD across several southern European countries, including Spain, Portugal, Italy, Greece, and Cyprus [58]. Some variations can be expected, probably due to the applied methodological framework and different instruments used for assessing adherence to the MD [55]. For example, one study from Spain showed a poor level of adherence to the MD in the general population and specific areas of Spain [59], while another one showed moderate adherence [60]. Nevertheless, deflection from a traditional MD diet and lifestyle represents a lost opportunity, not only from the perspective of achieving less-than-ideal individual and population health, but also from the perspective of environmental protection, possible degradation of sociocultural food values, and loss of positive local economic returns [61]. Additionally, a higher prevalence of adherence to the MD in the population can also serve as a safeguard from consumption of ultra-processed foods [62]. This was shown even in very young children from Spain, whose adherence to the traditional MD was inversely associated with energy intake from ultra-processed foods [63].

Previous studies have demonstrated that individual and contextual socio-economic factors are strong determinants of dietary habits and that poorer socio-economic groups are less likely to follow a healthy lifestyle [64]. On the other hand, social position in terms of education, occupational class, and income level represents a good predictor for healthy eating behavior [65,66]. People with a higher educational status have been shown to have a healthier consumption pattern [67]. Higher educational status was also associated with better nutritional intakes in lower GDP countries, while lower-income countries and lower education groups had poorer diets, particularly in terms of micronutrients intake [40].

The current economic and social European context—the increasing crisis, lack of jobs, various challenges due to the COVID-19 pandemic and consequent fall in income associated with cost inflation—could make people inclined to save money in all possible ways. In this context, the most exposed are the disadvantaged groups because they prefer buying food at low prices that are often of low quality [68]. Foods of lower nutritional value and lower-quality diets generally cost less per calorie and tend to be selected by groups of lower socioeconomic status [69]. On the other hand, people with low socio-economic status do not obtain the same health outcomes as those with high socio-economic status, even if both groups follow the same eating pattern [70]. Concretely, high adherence to the MD was associated with cardiovascular protection in higher but not in lower socio-economic groups from Italy, with a similar result observed for both education level and household income groups [70].

In some European countries, it was demonstrated that socio-economic status could modulate adherence to the MD [71,72]. For example, in a study carried out in the adult population from the Balearic Islands, people with a higher educational and socio-economic level showed higher rates of adherence to the Mediterranean pattern [57]. On the other hand, adherence to the MD in the South of Italy was found to be at low levels due to poor knowledge on MD concerning its beneficial effects [73], whereas social status in France was important for healthy eating only through an interaction between level of education and area of residence [64]. A similar association between education and MD was observed in our previous study from Croatia, where less educated people had a reduced likelihood of being adherent to the MD [31]. On the other hand, our current study did not corroborate such a finding, probably due to the inclusion of additional socio-economic indicators in the analysis (subjective and objective material status). Hence, we have identified only a significant association between overall adherence to the MD and objective material status. Subjects reporting the highest objective material status (fourth quartile) demonstrated a 93% higher probability of adhering to the MD than those belonging to the first quartile of objective material status, with similar findings for subjects within the second and third quartile groups (38% and 29%, respectively). This was in line with previous results, where higher household income was positively associated with greater adherence to the MD [39,74].

Interestingly, subjects with a higher educational attainment had a greater probability for appropriate adherence to dairy products, potatoes, and red meat intake recommendations, but they exhibited lesser adherence to cereals, olive oil, legumes, fish, and white meat intake recommendations. This represents a considerable departure from the traditional MD pattern. For example, Biesbroek et al. revealed that people with low education consumed more potatoes, whereas highly educated people consumed more olive oil and fish [75]. Another study showed that highly educated people in Italy also consumed white meat slightly less than in the past [56], while Bonaccio et al. had a similar conclusion for the consumption of white meat, which was again opposite when it came to people with high educational status and consumption of olive oil and fish [70]. Similarly, higher educational status was shown to be positively associated with fish intake [76]. Other MD food components were equally consumed by all educational groups in our sample, as previously shown in another study by Bonaccio et al. [39].

In general, highly educated people have a higher income, and they tend to follow MD recommendations [66]. This could be explained by the fact that greater adherence to the Mediterranean diet was associated with higher dietary cost, which might represent a barrier to healthy eating [35]. For instance, in a study including a representative national sample of 3534 children and young people from Spain, researchers have found that high adherence to the MD was more expensive than low adherence by 0.71 Euros per day [37].

Interestingly, we failed to find any association between subjective material status and adherence to the MD, whereas objective material status presented as the most prominent socio-economic indicator for overall adherence to the MD and for several food groups. For example, subjects in the higher quartiles of material status had higher adherence to fruit, vegetables, olive oil, and fish intake recommendations, but also lower adherence to red meat and sweets intake. Interestingly, olive oil and fish intake had an opposing contribution of educational level and material status to their adherence, such that lower education and higher material status were both associated with greater adherence. This could be explained by the fact that older, less educated people from Dalmatia, especially from remote islands, still tend to produce their own olive oil, and they catch fish on their own, which could be behind their higher intake of these foods (statement based on personal communication with subjects included in the study).

Our results are largely in line with previous studies, which showed that female gender and non-smokers [77,78,79], older adults [77,80,81], and more physically active people displayed higher adherence to the MD pattern, while higher body mass index was generally associated with lower adherence to the MD [78,79,80,82]. Our results partially replicated such associations, as subjects with higher levels of physical activity had up to a 50% greater probability of being adherent to the MD in comparison to the ones with light activity, while BMI was not significantly associated with adherence to the MD in our overall sample. This is in contrast to some previous findings [72,83,84], but in line with some studies [85,86]. These differences between previous results are probably due to the employed study design (cross-sectional vs. longitudinal, observational vs. experimental design), and characteristics of included subjects (primarily age and health status), leaving the association between adherence to the MD and BMI a topic for further investigation and open discussion.

The effect of the economic crisis of 2007–2008 on the adherence to the MD was a topic of several previous studies. For instance, it was found that adherence to the MD was lower in subjects from Italy reporting a negative impact of the crisis on their diet [87]. Additionally, the prevalence of adherence to MD among southern Italian citizens enrolled within the Moli-sani study was 31.3% during the 2005–2006 period, which dramatically fell during 2007–2010 (18.3%), most strongly affecting elderly, less affluent people, and urban areas dwellers [88]. Our results also revealed decreased odds for adherence to different food groups, i.e., adherence odds for vegetables, cereals, fruit, fish, legumes, dairy products, potatoes, and olive oil. On the other hand, odds of adherence to red meat and sweets recommendations increased after the recession.

Similar findings were demonstrated in Portugal, where a significant decrease in consumption of fish, fruit and vegetables was recorded from 2005/2006 to 2014 [29]. A cross-sectional study from Greece showed that parents who reported that the financial crisis affected their food spending also reported lower consumption of fruits, carbohydrate foods, and legumes, and increased intake of nutrient-poor/energy-dense foods, while their children had reduced weekly consumption of vegetables and increased weekly consumption of nutrient-poor/energy-dense foods [89]. These and other recent evidence show a possible involvement of the economic crisis, and material resources as strong determinants of adherence to the MD in the period after the recession started [90], given that a direct positive association between the cost of the diet and adherence to the MD has been established [36]. However, it is hard to distinguish the contribution of recession due to the economic crisis from the impact of the steady process of westernization of traditional dietary habits, including MD. For instance, it was noted by FAO that “the Mediterranean region is passing through a ‘nutritional transition’ in which problems of undernutrition coexist with overweight, obesity and food related chronic diseases” [91]. For example, an ecological study of the changes in food patterns in Europe over the last 40 years revealed that the greatest changes have occurred in Mediterranean Europe [92]. For instance, an increase of 20% in total energy availability was noted, alongside with a 48% increase in energy availability from lipids, and 20% decrease from carbohydrates, with a significant fall in the energy supplied by cereals (30%) and wine (55%), while the contribution of milk and dairy products increased by 78% and 24%, respectively [92]. For example, it was estimated that the Spanish diet shifted away from the traditional MD, now containing three times more meat, dairy and sugar products, and a third fewer fruits, vegetables, and cereals [93]. In our sample available for follow-up, we have detected similar deviations. For example, to our great dismay, vegetables adherence was reduced by 35%, followed by a reduction in fish adherence by 23%, white meat by 12%, cereals by 11%, and dairy products by 10%, while fruit adherence was reduced by only 2%. However, we did record a few positive trends, such as an increase in adherence for nuts (128%), sweets (113%; denoting reduced intake), red meat (56%; also denoting reduced intake), and wine (50%). Overall, the adherence to the MD remained stable, which was probably a consequence of differences in specific MD food constituents.

As already mentioned, a continuous increase in red and processed meat has been observed over the last couple of decades, while fruit, cereals, and vegetable consumption has decreased in different countries [21,94,95]. Our findings are in line with these trends, except for red meat intake, for which compliance was improved. To our satisfaction, an improvement over time was also recorded for sweets adherence in our study, which was in contrast to the findings from Portugal, where sweets/desserts consumption was significantly higher in 2014 compared to 2005/2006 [29]. However, a similar decreased trend for sweets intake were observed in Northern Italy [56], and Norway, Sweden, and Finland [96]. A study conducted among adults in Lebanon showed a decrease in the consumption of bread, fruits, fresh fruit juices, milk and eggs, whereas the consumption of added fats and oils, poultry, cereals and cereal-based products, chips and salty crackers, sweetened milk and hot beverages increased over time [97]. These findings indicate slightly different, yet similar patterns of change in different populations. The important next step in any effort to improve dietary habits in communities is the identification of factors associated with such changes, in order to be able to implement targeted interventions. We have conducted such an analysis, which pointed to several characteristics associated with the change in adherence to the MD over time. Female gender, older age at baseline, the highest level of education, and a moderate level of physical activity were positively associated with MDSS change during follow-up in a multivariate model. On the other hand, the MDSS score at baseline was negatively associated with the MDSS change during the follow-up, indicating that people with a higher baseline adherence to the MD tended to recede over time, while those with lower adherence strived toward increasing adherence to the MD. These findings highlight a continuous change of dietary patterns in the population, requiring constant monitoring of trends and identification of the drivers of such change. This is relevant from the perspective of population health and delivery of adequate health care, as well as from the perspective of economic, social and cultural development.

The importance of a healthy lifestyle and healthy dietary habits came to the frontline of attention due to the COVID-19 pandemic. For example, preliminary findings from the ecological study showed that Mediterranean diet adherence was negatively associated with both COVID-19 cases and related deaths in Spain and 23 other OECD countries, which the authors attributed to the anti-inflammatory properties of the Mediterranean diet [98]. On the other hand, an unhealthy lifestyle and associated metabolic disturbances and concomitant chronic diseases were shown to increase the risk for adverse outcomes after SARS-CoV-2 infection [99].

The traditional Mediterranean diet was shown to be beneficial in the prevention of weight gain and abdominal obesity [42]. On the other hand, a lower educational level was often found to be associated with a higher prevalence of overweight and obesity [39,100]—the same as economic affluence at a country level, reflecting a potential adverse outcome concomitant with economic growth [101]. While the relationship between socio-economic status and health outcomes was frequently emphasized for the Mediterranean area [100], the synergy between those two determinants was not substantially investigated in the population of Southern Croatia. Our results indicate that the average BMI had increased from 25.76 kg/m^2^ at baseline to 27.44 kg/m^2^ during the follow-up period. This is consistent with the trend of increasing rates of obesity across 147 countries [101]. BMI change during follow-up was positively associated with female gender, and negatively with initial BMI, initial adherence to the MD, and with change in adherence to the MD, as found in the regression analysis. This means that people with a lower BMI at the beginning of the study tended to experience a rise in BMI, while those who started with higher values managed to diminish it over time. An encouraging finding is that individuals with a higher-level/score in MD adherence experienced lower BMI change or even its decrease.

An important limitation of our study that needs to be mentioned here is the use of the cross-sectional design for estimating the association between socio-economic status and adherence to the MD, which limits the inference on causality. However, we did employ an additional follow-up study design in order to confirm the initial findings and observe time trends, as well as to investigate the association between initial socio-economic status and change in adherence to the MD, and BMI change in our sample. Another limitation is the broad sampling period of subjects included in our study, which stretched from 2003 to 2015. In order to control for the effect of the study period in the logistic regression analysis, we included the actual follow-up time as one of the predictor variables, as well as the variable “economic crisis of 2007–2008”. We also managed to obtain a smaller than ideal sample size and lesser response rate in the follow-up study (28.7%), due to the older age of subjects and their inability to participate in the follow-up examination. Advantages of the study include a relatively large overall sample size, inclusion of many potential predictors, and sampling from the general population of inhabitants from the Mediterranean region of Croatia. Determinants of Mediterranean diet adherence were so far only marginally investigated in the population of Dalmatia in Croatia, and we are filling this gap.

In conclusion, this is the first study from Croatia to examine the changes in adherence to the MD over time. Additionally, we have identified several important characteristics associated with greater adherence to the MD and with its change over time. These insights should be used to inform the necessary and targeted interventions aimed at increasing MD uptake in order to ensure beneficial outcomes. These include, but are not limited to, the promotion and advancement of individual and population health, ensuring environmental sustainability, and positive impacts on local economies and tourism, as well as the very important outcome of the preservation of cultural heritage for generations to come.

## Figures and Tables

**Table 1:** Demographic characteristics and adherence to the MD (14 food components and overall adherence expressed as MDSS ≥ 14 points), according to the place of residence in a total sample of 4671 subjects.

	Island of Vis N = 1012	Island of Korčula N = 2651	City of Split N = 1008	Overall *p* (Pairwise Comparison *p* Values)
Sex; *n* (%)				0.011 (0.003 ^V-K^, 0.168 ^V-S^, 0.196 ^K-S^)
Men	423 (41.8)	967 (36.5)	391 (38.8)	
Women	589 (58.2)	1684 (63.5)	617 (61.2)	
Age (years); median (IQR)	56.00 (24.00)	55.00 (23.25)	52.00 (21.00)	<0.001 (<0.001 ^V-K^, <0.001 ^V-S^, <0.001 ^K-S^)
Education (years of schooling); median (IQR)	11.00 (4.00)	12.00 (3.00)	12.00 (4.00)	<0.001 (<0.001 ^V-K^, <0.001 ^V-S^, <0.001 ^K-S^)
Subjective material status; median (IQR)	3.00 (0.00)	3.00 (1.00)	3.00 (1.00)	<0.001 (<0.001 ^V-K^, <0.001 ^V-S^, <0.001 ^K-S^)
Objective material status; median (IQR)	10.00 (5.00)	10.00 (3.00)	12.00 (3.00)	<0.001 (<0.001 ^V-K^, <0.001 ^V-S^, <0.001 ^K-S^)
Body mass index (kg/m^2^); median (IQR)	27.08 (6.05)	24.59 (5.94)	26.60 (5.63)	<0.001 (<0.001 ^V-K^, 0.024 ^V-S^, <0.001 ^K-S^)
Chronic diseases *; *n* (%)				<0.001 (0.011 ^V-K^, <0.001 ^V-S^, <0.001 ^K-S^)
None	542 (53.6)	1565 (59.0)	677 (67.2)	
1	289 (28.6)	677 (25.5)	248 (24.6)	
≥2	181 (17.9)	409 (15.4)	83 (8.2)	
Smoking (pack years); median (IQR)	0.00 (10.00)	0.00 (3.00)	0.00 (3.00)	0.004 (0.002 ^V-K^, 0.007 ^V-S^, 0.804 ^K-S^)
Smoking; *n* (%)				<0.001 (0.001 ^V-K^, 0.105 ^V-S^, 0.005 ^K-S^)
current smokers	288 (28.5)	741 (28.0)	266 (26.5)	
ex-smokers	303 (30.0)	584 (22.2)	275 (27.4)	
never-smokers	419 (41.5)	1306 (49.6)	464 (46.2)	
Physical activity; *n* (%)				<0.001 (<0.001 ^V-K^, <0.001 ^V-S^, <0.001 ^K-S^)
light	264 (26.2)	537 (20.5)	358 (35.6)	
moderate	580 (57.5)	1815 (69.2)	610 (60.7)	
intensive	164 (16.3)	271 (10.3)	37 (3.7)	
MDSS; median (IQR)	12.00 (5.00)	11.00 (6.00)	12.00 (5.00)	<0.001 (<0.001 ^V-K^, 0.554 ^V-S^, 0.001 ^K-S^)
MDSS according to age group; median (IQR)				
18.0–34.9	10.00 (5.00)	9.00 (5.00)	10.00 (5.00)	0.009 (0.004 ^V-K^, 0.225 ^V-S^, 0.068 ^K-S^)
35.0–64.9	12.00 (5.00)	11.00 (6.00)	12.00 (5.00)	0.001 (0.004 ^V-K^, 0.982 ^V-S^, 0.003 ^K-S^)
≥65.0	12.00 (5.00)	12.00 (6.00)	13.00 (5.00)	<0.001 (0.009 ^V-K^, <0.001 ^V-S^, <0.001 ^K-S^)
MDSS components adherence; *n* (%)				
fruit	596 (58.9)	1399 (52.8)	636 (63.1)	<0.001 (0.001 ^V-K^, 0.053 ^V-S^, <0.001 ^K-S^)
vegetables	439 (43.4)	980 (37.0)	418 (41.5)	0.001 (<0.001 ^V-K^, 0.385 ^V-S^, 0.012 ^K-S^)
cereals	986 (97.4)	2367 (89.3)	929 (92.2)	<0.001 (<0.001 ^V-K^, <0.001 ^V-S^, 0.009 ^K-S^)
olive oil	586 (57.9)	1835 (69.2)	643 (63.8)	<0.001 (<0.001 ^V-K^, 0.007 ^V-S^, 0.002 ^K-S^)
nuts	27 (2.7)	117 (4.4)	71 (7.0)	<0.001 (0.015 ^V-K^, <0.001 ^V-S^, 0.001 ^K-S^)
dairy products	256 (25.3)	592 (22.3)	270 (26.8)	0.010 (0.057 ^V-K^, 0.446 ^V-S^, 0.005 ^K-S^)
potatoes	686 (67.8)	1774 (66.9)	823 (81.6)	<0.001 (0.617 ^V-K^, <0.001 ^V-S^, <0.001 ^K-S^)
legumes	326 (32.2)	714 (26.9)	252 (25.0)	0.001 (0.002 ^V-K^, <0.001 ^V-S^, 0.236 ^K-S^)
eggs	297 (29.3)	662 (25.0)	246 (24.4)	0.013 (0.007 ^V-K^, 0.012 ^V-S^, 0.723 ^K-S^)
fish	838 (82.8)	1769 (66.7)	692 (68.7)	<0.001 (<0.001 ^V-K^, <0.001 ^V-S^, 0.269 ^K-S^)
white meat	499 (49.3)	1077 (40.6)	381 (37.8)	<0.001 (<0.001 ^V-K^, <0.001 ^V-S^, 0.118 ^K-S^)
red meat	261 (25.8)	700 (26.4)	248 (24.6)	0.537 (0.705 ^V-K^, 0.539 ^V-S^, 0.266 ^K-S^)
sweets	181 (17.9)	808 (30.5)	168 (16.7)	<0.001 (<0.001 ^V-K^, 0.469 ^V-S^, <0.001 ^K-S^)
wine	204 (20.2)	459 (17.3)	177 (17.6)	0.124 (0.046 ^V-K^, 0.136 ^V-S^, 0.861 ^K-S^)
Adherence to the MD (MDSS ≥ 14 points); *n* (%)	315 (31.1)	711 (26.8)	306 (30.4)	0.012 (0.009 ^V-K^, 0.708 ^V-S^, 0.033 ^K-S^)
Adherence to the MD according to age group (MDSS ≥ 14 points); *n* (%)				
18.0–34.9 years	22 (20.0)	49 (12.3)	26 (14.8)	0.012 (0.026 ^V-K^, 0.745 ^V-S^, 0.070 ^K-S^)
35.0–64.9 years	158 (29.3)	393 (25.4)	203 (30.5)	
≥65.0 years	135 (37.2)	269 (38.4)	77 (46.1)	

IQR—interquartile range; MDSS—Mediterranean Diet Serving Score; MD—Mediterranean diet; *p* values for categorical variables were obtained with the chi-squared test, and for numerical variables with the Kruskal–Wallis test. Pairwise comparison *p* values for categorical variables were obtained with the chi-squared test, and for numerical variables with Mann–Whitney U test. * chronic diseases included any or more than one of the following diagnoses: hypertension, diabetes, CHD, CVI, cancer, bipolar disorder, hyperlipidemia and gout. ^V-K^ Pairwise comparison *p* value: Island of Vis vs. Island of Korčula. ^V-S^ Pairwise comparison *p* value: Island of Vis vs. City of Split. ^K-S^ Pairwise comparison *p* value: Island of Korčula vs. City of Split.

**Table 2:** Characteristics associated with adherence to the MD (MDSS ≥ 14 points) in the total sample (N = 4671), as determined by the logistic regression analysis.

	Unadjusted Odds Ratio (95% Confidence Interval); *p*	Adjusted Odds Ratio (95% Confidence Interval); *p*
Sex		
Male; Ref.	1.00	1.00
Female	1.60 (1.39, 1.83); <0.001	1.85 (1.58, 2.17); <0.001
Age group		
18–34.9; Ref.	1.00	1.00
35–64.9	2.29 (1.82, 2.88); <0.001	1.99 (1.54, 2.57); <0.001
≥65.0	3.89 (3.05, 4.97); <0.001	3.81 (2.83, 5.12); <0.001
Place of residence		
City of Split; Ref.	1.00	1.00
Island of Vis	1.04 (0.86, 1.25); 0.708	1.04 (0.84, 1.29); 0.696
Island of Korčula	0.84 (0.72, 0.99); 0.033	1.63 (1.31, 2.02); <0.001
Education (Years of schooling)		
elementary (0–8); Ref.	1.00	1.00
high school (9–12)	0.69 (0.78, 0.80); <0.001	0.93 (0.77, 1.14); 0.492
higher (13+)	0.94 (0.79, 1.12); 0.494	1.19 (0.95, 1.5); 0.130
Subjective material status		
worse than average; Ref.	1.00	1.00
average	1.13 (0.92, 1.39); 0.250	1.14 (0.91, 1.44); 0.258
better than average	1.28 (1.03, 1.61); 0.028	1.16 (0.89, 1.51); 0.267
Objective material status		
1st quartile; Ref.	1.00	1.00
2nd quartile	1.12 (0.94, 1.35); 0.216	1.38 (1.12, 1.70); 0.002
3rd quartile	0.98 (0.82, 1.17); 0.791	1.29 (1.04, 1.61); 0.020
4th quartile	1.52 (1.27, 1.83); <0.001	1.93 (1.53, 2.43); <0.001
Chronic diseases *		
≥2; Ref.	1.00	1.00
1	0.85 (0.69, 1.04); 0.107	0.93 (0.75, 1.17); 0.546
none	0.69 (0.57, 0.82); <0.001	0.93 (0.75, 1.16); 0.507
Smoking		
current smokers; Ref.	1.00	1.00
ex-smokers	1.70 (1.41, 2.03); <0.001	1.40 (1.14, 1.71); 0.001
never-smokers	1.75 (1.49, 2.06); <0.001	1.36 (1.13, 1.63); 0.001
Physical activity		
light; Ref.	1.00	1.00
moderate	1.24 (1.07, 1.45); 0.005	1.44 (1.21, 1.70); <0.001
intensive	1.16 (0.91, 1.48); 0.222	1.50 (1.15, 1.97); 0.003
Body mass index category ^#^		
18.0–24.9 (kg/m^2^); Ref.	1.00	1.00
25.0–29.9 (kg/m^2^)	0.92 (0.79, 1.05); 0.218	0.98 (0.83, 1.16); 0.834
≥30.0 (kg/m^2^)	0.79 (0.66, 0.95); 0.013	0.84 (0.68, 1.05); 0.123
The economic crisis of 2007–2008		
before; Ref.	1.00	1.00
after	0.40 (0.35, 0.46); <0.001	0.31 (0.25, 0.38); <0.001

Adjusted odds ratios, 95% confidence intervals and *p* values were calculated using a multivariate logistic regression model simultaneously adjusted for all the covariates listed in this table (enter method). * chronic diseases included any or more than one of the following diagnoses: hypertension, diabetes, CHD, CVI, cancer, bipolar disorder, hyperlipidemia and gout; **^#^** 53 subjects with BMI < 18.5 kg/m^2^ were excluded from the analysis due to small sample size of the group and negative impact on the model performance.

**Table 3:** Adherence to 14 MD food groups and the overall MD (MDSS ≥ 14 points) at baseline and at the follow-up (N = 1342; 366 subjects from Korčula, 494 from Split, and 482 subjects from Vis).

	Baseline N = 1342	Follow Up N = 1342	Percent Change (%)	*p*
Sex; *n* (%)				
men	503 (37.5)	-	-	-
women	839 (62.5)			
Age (years); median (IQR)	55.00 (18.00)	62.01 (16.96)	-	-
Age group; *n* (%)				-
18.0–34.99	127 (9.5)	58 (4.3)	-	
35.0–64.99	926 (69.0)	724 (53.9)		
65+	289 (21.5)	560 (41.7)		
Body mass index (kg/m^2^); median (IQR)	25.76 (5.74)	27.44 (5.06)	6.5	<0.001
Adherence to the MD (MDSS ≥ 14 points); *n* (%)	491 (36.6)	449 (33.5)	−8.5	0.056
MDSS components adherence; *n* (%)				
fruit	868 (64.7)	848 (63.2)	−2.3	0.341
vegetables	643 (47.9)	417 (31.1)	−35.1	<0.001
cereals	1277 (95.2)	1138 (84.8)	−10.9	<0.001
potatoes	985 (73.4)	1183 (88.2)	20.2	<0.001
olive oil	893 (66.5)	927 (69.1)	3.9	0.112
nuts	68 (5.1)	156 (11.6)	127.5	<0.001
dairy products	356 (26.5)	309 (23.0)	−9.6	0.030
legumes	400 (29.8)	457 (34.1)	14.4	0.011
eggs	339 (25.3)	405 (30.2)	19.4	0.002
fish	1036 (77.2)	793 (59.1)	−23.4	<0.001
white meat	556 (41.4)	487 (36.3)	−11.6	0.005
red meat	347 (25.9)	544 (40.5)	56.4	<0.001
sweets	276 (20.6)	588 (43.8)	112.6	<0.001
wine	268 (20.0)	403 (30.0)	50.0	<0.001

MDSS—Mediterranean Diet Serving Score. MD—Mediterranean Diet. *p* values for categorical variables were obtained using McNemar test and for numerical using Wilcoxon Signed-Ranks Test.

**Table 4:** Characteristics associated with the absolute change in the Mediterranean Diet Serving Score (MDSS) and the BMI across the follow-up period, as determined by the linear regression model (sample size is 1342 subjects; all independent variables were included in the model simultaneously).

	MDSS Change during Follow-Up Beta (95% Confidence Interval); *p*	BMI Change during Follow-Up Beta (95% Confidence Interval); *p*
Sex		
Male; Ref.	1.00	1.00
Female	0.41 (0.00, 0.83); 0.049	0.33 (0.06, 0.60); 0.016
Age at baseline (years)	0.05 (0.03, 0.06); <0.001	−0.01 (−0.02, 0.00); 0.192
Follow up time (years)	0.03 (−0.09, 0.16); 0.571	−0.07 (−0.15, 0.01); 0.099
Place of residence		
City of Split; Ref.	1.00	1.00
Island of Vis	0.28 (−0.75, 1.32); 0.590	0.86 (0.18, 1.54); 0.013
Island of Korčula	0.00 (−0.81, 0.81); 0.992	3.68 (3.15, 4.21); <0.001
Education (years of schooling)		
elementary (0–8); Ref.	1.00	1.00
high school (9–12)	−0.11 (−0.67, 0.46); 0.708	−0.05 (−0.42, 0.32); 0.799
higher (13+)	0.71 (0.07, 1.36); 0.031	0.06 (−0.36, 0.48); 0.769
Subjective material status		
worse than average; Ref.	1.00	1.00
average	0.05 (−0.59, 0.69); 0.872	0.15 (−0.26, 0.57); 0.468
better than average	−0.01 (−0.72, 0.71); 0.982	0.11 (−0.36, 0.57); 0.655
Objective material status		
1st quartile; Ref.	1.00	1.00
2nd quartile	−0.14 (−0.71, 0.43); −0.637	0.07 (−0.30, 0.44); 0.724
3rd quartile	0.02 (−0.56, 0.59); 0.955	0.06 (−0.31, 0.44); 0.733
4th quartile	−0.06 (−0.68, 0.56); 0.859	0.02 (−0.38, 0.42); 0.921
Chronic diseases *		
≥2; Ref.	1.00	1.00
1	−0.23 (−0.91, 0.44); 0.497	0.24 (−0.19, 0.68); 0.276
none	−0.02 (−0.68, 0.64); 0.946	0.26 (−0.17, 0.69); 0.240
Smoking		
current smokers; Ref.	1.00	1.00
ex-smokers	−0.20 (−0.74, 0.34); 0.468	−0.07 (−0.42, 0.28); 0.707
never-smokers	0.12 (−0.38, 0.62); 0.632	0.02 (−0.31, 0.34); 0.909
Physical activity		
light; Ref.	1.00	1.00
moderate	0.72 (0.27, 1.16); 0.002	−0.02 (−0.31, 0.27); 0.887
intensive	0.69 (−0.02, 1.40); 0.057	0.23 (−0.24, 0.70); 0.331
BMI at baseline (kg/m^2^)	−0.03 (−0.09, 0.02); 0.188	−0.11 (−0.14, −0.07); <0.001
MDSS at baseline	−0.64 (−0.70, −0.58); <0.001	−0.07 (−0.12, −0.03); 0.001
MDSS change during follow-up	-	−0.04 (−0.07, 0.00); 0.041

* Chronic diseases included any or more than one of the following diagnoses: hypertension, diabetes, CHD, CVI, cancer, bipolar disorder, hyperlipidemia and gout. MDSS—Mediterranean Diet Serving Score.

## Data Availability

The datasets generated during and/or analyzed during the current study are available from the corresponding author upon reasonable request.

## Supplementary Materials

The following are available online at https://www.mdpi.com/article/10.3390/nu13113802/s1, Table S1: Characteristics associated with adherence to the main food groups within the Mediterranean diet serving score (MDSS), as determined by the multivariate logistic regression analysis (N = 4671).
