# Supplementary material for: The Role of Socioeconomic Status in Adherence to the Mediterranean Diet and Body Mass Index Change: A Follow-Up Study in the General Population of Southern Croatia
Source: Nutrients. 2021 Oct 26;13(11):3802. doi: 10.3390/nu13113802 (PMC8624926; doi:10.3390/nu13113802)
Supplement: Supplementary file 1 [file nutrients-13-03802-s001.zip › nutrients-1403254-supplementary.pdf]

nutrients

MDPI

Supplementary Table S1. Characteristics associated with adherence to the main food groups within the Mediterranean diet serving score (MDSS), as determined by the multivariate logistic regression analysis (N = 4,671)

	**Fruit** aOR (95% CI); *P*	**Vegetables** aOR (95% CI); *P*	**Cereals** aOR (95% CI); *P*	**Olive oil** aOR (95% CI); *P*	**Nuts** aOR (95% CI); *P*	**Dairy** aOR (95% CI); *P*	**Potatoes** aOR (95% CI); *P*	**Legumes** aOR (95% CI); *P*	**Eggs** aOR (95% CI); *P*	**Fish** aOR (95% CI); *P*	**White meat** aOR (95% CI); *P*	**Red meat** aOR (95% CI); *P*	**Sweets** aOR (95% CI); *P*	**Wine** aOR (95% CI); *P*
**Sex**														
Male; Ref.	1.00	1.00	1.00	1.00	1.00	1.00	1.00	1.00	1.00	1.00	1.00	1.00	1.00	1.00
Female	2.42 (2.10, 2.78); <0.001	1.91 (1.55, 2.06); <0.001	0.82 (0.64, 1.05); 0.110	1.22 (1.06, 1.40); 0.007	1.97 (1.40, 2.78); <0.001	1.59 (1.35, 1.86); <0.001	0.97 (0.83, 1.12); 0.652	1.13 (0.98, 1.31); 0.102	0.82 (0.71, 0.95); 0.009	1.08 (0.93, 1.26); 0.286	1.04 (0.91, 1.19); 0.520	1.96 (1.67, 2.30); <0.001	1.00 (0.85, 1.17); 0.981	0.39 (0.33, 0.46); <0.001
**Age group** (years)														
18-34 Ref.	1.00	1.00	1.00	1.00	1.00	1.00	1.00	1.00	1.00	1.00	1.00	1.00	1.00	1.00
35-64.9	1.61 (1.32, 1.96); <0.001	1.56 (1.27, 1.93); <0.001	1.44 (1.06, 1.96); 0.020	1.43 (1.18, 1.73); <0.001	1.35 (0.82, 2.20); 0.235	0.98 (0.78, 1.21); 0.823	0.58 (0.46, 0.73); <0.001	1.01 (0.82, 1.26); 0.901	0.84 (0.69, 1.03); 0.091	1.32 (1.08, 1.61); 0.006	1.01 (0.84, 1.22); 0.923	1.23 (0.98, 1.56); 0.075	1.90 (1.45, 2.49); <0.001	1.69 (1.29, 2.22); <0.001
≥65.0	2.83 (2.21, 3.62); <0.001	2.33 (1.81, 3.00); <0.001	1.40 (0.94, 2.09); 0.101	2.40 (1.87, 3.07); <0.001	1.92 (1.09, 3.40); 0.024	1.24 (0.95, 1.63); 0.117	0.75 (0.59, 0.95); 0.018	1.29 (0.99, 1.67); 0.055	0.63 (0.48, 0.81); <0.001	2.01 (1.55, 2.60); <0.001	0.90 (0.71, 1.14); 0.370	1.58 (1.20, 2.09); 0.001	2.42 (1.78, 3.29); <0.001	2.41 (1.76, 3.31); <0.001
**Place of residence**														
City of Split; Ref.	1.00	1.00	1.00	1.00	1.00	1.00	1.00	1.00	1.00	1.00	1.00	1.00	1.00	1.00
Island of Vis	0.84 (0.68, 1.02); 0.083	1.08 (0.89, 1.32); 0.419	2.76 (1.72, 4.41); <0.001	0.70 (0.58, 0.86); 0.001	0.46 (0.28, 0.75); 0.002	1.13 (0.91, 1.41); 0.254	0.64 (0.51, 0.80); <0.001	1.22 (0.99, 1.51); 0.068	1.40 (1.13, 1.74); 0.002	2.16 (1.72, 2.71); <0.001	1.40 (1.16, 1.70); 0.001	1.03 (0.82, 1.29); 0.804	0.83 (0.64, 1.07); 0.144	1.27 (0.99, 1.63); 0.057

Island of Korčula	0.98 (0.79, 1.22); 0.849	1.63 (1.33, 2.00); <0.001	1.10 (0.74, 1.64); 0.629	1.53 (1.22, 1.91); <0.001	0.67 (0.43, 1.04); 0.072	1.14 (0.91, 1.43); 0.258	0.58 (0.48, 0.70); <0.001	1.54 (1.24, 1.92); <0.001	0.98 (0.78, 1.24); 0.880	1.44 (1.14, 1.81); 0.002	1.08 (0.88, 1.32); 0.457	0.89 (0.70, 1.12); 0.319	1.82 (1.42, 2.34); <0.001	1.08 (0.83, 1.40); 0.561
**Education (years of schooling)**														
elementary (0-8); Ref.	1.00	1.00	1.00	1.00	1.00	1.00	1.00	1.00	1.00	1.00	1.00	1.00	1.00	1.00
high school (9- 12)	1.17 (0.98, 1.41); 0.085	1.06 (0.89, 1.27); 0.521	0.74 (0.53, 1.04); 0.081	0.66 (0.54, 0.79); <0.001	1.07 (0.69, 1.66); 0.760	1.07 (0.87, 1.31); 0.535	1.43 (1.19, 1.71); <0.001	0.72 (0.60, 0.87); 0.001	1.04 (0.86, 1.27); 0.675	0.77 (0.63, 0.94); 0.011	0.76 (0.64, 0.90); 0.002	1.14 (0.94, 1.40); 0.188	1.05 (0.86, 1.28); 0.653	1.12 (0.89, 1.41); 0.324
higher (13+)	1.20 (0.97, 1.49); 0.092	1.16 (0.94, 1.44); 0.171	0.60 (0.41, 0.88); 0.010	0.69 (0.55, 0.86); 0.001	1.61 (1.00, 2.59); 0.051	1.32 (1.04, 1.67); 0.022	2.41 (1.92, 3.03); <0.001	0.80 (0.64, 0.99); 0.045	0.92 (0.73, 1.16); 0.487	0.78 (0.62, 0.99); 0.040	0.73 (0.59, 0.89); 0.002	1.89 (1.50, 2.38); <0.001	1.01 (0.79, 1.28); 0.943	1.25 (0.96, 1.62); 0.101
**Subjective material status**														
worse than average; Ref.	1.00	1.00	1.00	1.00	1.00	1.00	1.00	1.00	1.00	1.00	1.00	1.00	1.00	1.00
average	1.23 (1.00, 1.51); 0.050	1.01 (0.83, 1.25); 0.894	0.88 (0.60, 1.28); 0.500	1.17 (0.95, 1.43); 0.135	1.22 (0.71, 2.10); 0.470	1.09 (0.86, 1.38); 0.461	1.05 (0.85, 1.30); 0.633	1.04 (0.84, 1.30); 0.690	0.74 (0.59, 0.91); 0.005	1.07 (0.86, 1.34); 0.517	1.02 (0.84, 1.25); 0.812	0.81 (0.66, 1.01); 0.059	0.89 (0.72, 1.12); 0.324	1.19 (0.91, 1.56); 0.209
better than average	1.20 (0.95, 1.52); 0.126	1.02 (0.81, 1.29); 0.853	0.82 (0.54, 1.25); 0.364	1.27 (1.01, 1.61); 0.045	1.75 (0.98, 3.12); 0.058	1.10 (0.84, 1.43); 0.499	1.22 (0.96, 1.56); 0.106	1.02 (0.80, 1.31); 0.861	0.76 (0.59, 0.97); 0.029	1.12 (0.87, 1.44); 0.369	0.98 (0.78, 1.22); 0.845	0.68 (0.53, 0.87); 0.002	0.90 (0.69, 1.16); 0.402	1.12 (0.83, 1.52); 0.459

**Objective material status**														
1st quartile; Ref.	1.00	1.00	1.00	1.00	1.00	1.00	1.00	1.00	1.00	1.00	1.00	1.00	1.00	1.00
2nd quartile	1.24 (1.03, 1.50); 0.025	1.20 (0.99, 1.45); 0.057	1.39 (1.00, 1.91); 0.048	1.35 (1.12, 1.63); 0.002	1.21 (0.78, 1.87); 0.387	1.11 (0.89, 1.37); 0.349	0.87 (0.71, 1.06); 0.158	1.18 (0.97, 1.43); 0.103	1.08 (0.88, 1.33); 0.445	1.43 (1.17, 1.74); <0.001	1.00 (0.84, 1.20); 0.967	0.73 (0.60, 0.89); 0.002	0.75 (0.62, 0.92); 0.007	0.99 (0.78, 1.26); 0.938
3rd quartile	1.28 (1.06, 1.56); 0.011	1.36 (1.12, 1.65); 0.002	1.31 (0.95, 1.81); 0.102	1.49 (1.23, 1.81); <0.001	0.74 (0.46, 1.19); 0.216	1.11 (0.89, 1.37); 0.349	0.73 (0.60, 0.89); 0.002	1.15 (0.94, 1.40); 0.187	1.14 (0.92, 1.4); 0.232	1.72 (1.41, 2.11); <0.001	0.92 (0.77, 1.10); 0.367	0.65 (0.53, 0.80); <0.001	0.63 (0.51, 0.78); <0.001	1.04 (0.81, 1.32); 0.778
4th quartile	1.57 (1.27, 1.94); <0.001	1.66 (1.34, 2.05); <0.001	1.30 (0.92, 1.84); 0.140	2.27 (1.83, 2.83); <0.001	1.35 (0.86, 2.14); 0.195	1.32 (1.05, 1.67); 0.019	0.80 (0.64, 1.00); 0.050	1.04 (0.83, 1.30); 0.737	1.18 (0.94, 1.48); 0.160	2.23 (1.78, 2.79); <0.001	0.93 (0.76, 1.13); 0.460	0.62 (0.50, 0.78); <0.001	0.70 (0.55, 0.88); 0.003	1.29 (1.00, 1.67); 0.054
**Chronic diseases***														
≥2; Ref.	1.00	1.00	1.00	1.00	1.00	1.00	1.00	1.00	1.00	1.00	1.00	1.00	1.00	1.00
1	0.92 (0.74, 1.14); 0.465	0.94 (0.76, 1.15); 0.529	1.50 (1.04, 2.17); 0.030	0.99 (0.79, 1.24); 0.923	1.25 (0.77, 2.05); 0.370	0.94 (0.74, 1.20); 0.622	0.94 (0.76, 1.17); 0.573	1.05 (0.84, 1.31); 0.681	1.06 (0.84, 1.35); 0.619	1.10 (0.87, 1.38); 0.436	1.00 (0.81, 1.22); 0.964	0.97 (0.78, 1.21); 0.782	0.70 (0.56, 0.86); 0.001	1.21 (0.94, 1.58); 0.144
none	0.86 (0.70, 1.06); 0.156	0.91 (0.74, 1.11); 0.341	1.33 (0.94, 1.90); 0.111	0.88 (0.71, 1.09); 0.241	1.13 (0.69, 1.85); 0.614	1.13 (0.90, 1.43); 0.303	1.10 (0.89, 1.36); 0.380	1.09 (0.87, 1.35); 0.462	1.24 (0.98, 1.57); 0.073	1.06 (0.84, 1.33); 0.641	0.96 (0.79, 1.17); 0.700	0.73 (0.59, 0.92); 0.006	0.53 (0.43, 0.66); <0.001	1.29 (1.00, 1.66); 0.052
**Smoking**														
current smokers; Ref.	1.00	1.00	1.00	1.00	1.00	1.00	1.00	1.00	1.00	1.00	1.00	1.00	1.00	1.00
ex, smokers	1.87 (1.56, 2.24); <0.001	1.04 (0.87, 1.25); 0.650	1.12 (0.82, 1.54); 0.475	1.26 (1.05, 1.51); 0.014	1.52 (0.98, 2.37); 0.062	1.02 (0.83, 1.25); 0.878	1.09 (0.89, 1.32); 0.402	1.01 (0.83, 1.22); 0.943	0.85 (0.70, 1.03); 0.096	1.29 (1.06, 1.58); 0.010	0.94 (0.79, 1.12); 0.519	1.22 (0.99, 1.50); 0.060	1.05 (0.86, 1.29); 0.635	1.18 (0.94, 1.47); 0.145
never, smokers	2.03 (1.73, 2.37); <0.001	1.01 (0.86, 1.18); 0.940	1.22 (0.94, 1.60); 0.137	1.12 (0.96, 1.31); 0.154	1.58 (1.07, 2.32); 0.021	1.09 (0.92, 1.30); 0.326	0.93 (0.78, 1.10); 0.378	0.98 (0.83, 1.16); 0.826	1.02 (0.86, 1.21); 0.803	1.05 (0.89, 1.23); 0.598	0.92 (0.79, 1.07); 0.293	1.22 (1.02, 1.46); 0.026	0.84 (0.70, 1.01); 0.064	1.05 (0.86, 1.29); 0.636

**Physical activity**														
light; Ref.	1.00	1.00	1.00	1.00	1.00	1.00	1.00	1.00	1.00	1.00	1.00	1.00	1.00	1.00
moderate	1.24 (1.07, 1.45); 0.005	1.43 (1.22, 1.67); <0.001	0.83 (0.63, 1.08); 0.169	1.29 (1.11, 1.50); 0.001	1.12 (0.79, 1.57); 0.524	0.98 (0.83, 1.16); 0.840	0.88 (0.75, 1.04); 0.140	1.50 (1.26, 1.77); <0.001	1.13 (0.96, 1.34); 0.152	1.33 (1.14, 1.56); <0.001	0.94 (0.81, 1.09); 0.395	0.99 (0.84, 1.16); 0.873	1.17 (0.98, 1.39); 0.085	0.93 (0.77, 1.13); 0.471
intensive	1.24 (0.97, 1.59); 0.081	1.75 (1.37, 2.23); <0.001	1.38 (0.83, 2.30); 0.220	1.48 (1.15, 1.90); 0.002	1.06 (0.58, 1.96); 0.842	0.84 (0.63, 1.12); 0.228	0.58 (0.45, 0.74); <0.001	1.61 (1.25, 2.08); <0.001	1.08 (0.83, 1.40); 0.570	1.87 (1.42, 2.48); <0.001	1.08 (0.86, 1.36); 0.521	0.70 (0.52, 0.93); 0.014	1.04 (0.79, 1.38); 0.775	0.72 (0.53, 0.98); 0.036
**Body mass index category[#]**														
18.0-24.9; Ref.	1.00	1.00	1.00	1.00	1.00	1.00	1.00	1.00	1.00	1.00	1.00	1.00	1.00	1.00
25.0-29.9	0.99 (0.85, 1.15); 0.850	0.99 (0.85, 1.15); 0.891	0.82 (0.63, 1.07); 0.147	0.94 (0.81, 1.10); 0.465	0.92 (0.66, 1.28); 0.629	0.92 (0.78, 1.09); 0.350	1.16 (0.99, 1.36); 0.076	1.03 (0.88, 1.21); 0.717	1.00 (0.85, 1.18); 0.990	1.09 (0.93, 1.28); 0.291	1.07 (0.93, 1.24); 0.339	1.04 (0.88, 1.23); 0.630	1.19 (1.00, 1.41); 0.049	0.89 (0.74, 1.07); 0.214
≥30.0	0.97 (0.80, 1.18); 0.780	0.97 (0.80, 1.18); 0.748	0.70 (0.51, 0.98); 0.037	0.85 (0.70, 1.04); 0.108	0.58 (0.36, 0.93); 0.025	0.99 (0.80, 1.23); 0.961	1.21 (0.98, 1.48); 0.070	1.17 (0.95, 1.43); 0.139	0.89 (0.72, 1.10); 0.264	0.97 (0.79, 1.19); 0.767	0.91 (0.76, 1.10); 0.328	0.84 (0.67, 1.04); 0.102	1.34 (1.08, 1.66); 0.009	0.80 (0.63, 1.02); 0.071
**The economic crisis of 2007–2008**														
before; Ref.	1.00	1.00	1.00	1.00	1.00	1.00	1.00	1.00	1.00	1.00	1.00	1.00	1.00	1.00
after	0.50 (0.41, 0.60); <0.001	0.32 (0.27, 0.39); <0.001	0.45 (0.32, 0.63); <0.001	0.71 (0.58, 0.86); 0.001	1.04 (0.68, 1.57); 0.864	0.64 (0.52, 0.78); <0.001	0.69 (0.57, 0.83); <0.001	0.53 (0.44, 0.64); <0.001	1.10 (0.89, 1.35); 0.371	0.51 (0.42, 0.63); <0.001	0.93 (0.77, 1.11); 0.397	1.39 (1.13, 1.71); 0.002	1.23 (1.01, 1.50); 0.043	0.97 (0.77, 1.22); 0.796

Aor—Adjusted odds ratios; 95% CI—95% confidence intervals; calculated using multivariate logistic regression model simultaneously adjusted for all the covariates listed in this table. * chronic diseases included any or more than one of the following diagnoses: hypertension, diabetes, CHD, CVI, cancer, bipolar disorder, hyperlipidemia and gout; [#] 53 subjects with BMI < 18.5 kg/m^2 were excluded from the analysis due to small sample size of the group and negative impact on the model performance.
